# TIAS (Timing, Individualised, Amalgamated, Stepwise) Algorithm for Efficacious and Cost-Effective Cardiac Monitoring to Detect Atrial Fibrillation After Stroke

**DOI:** 10.7759/cureus.91448

**Published:** 2025-09-01

**Authors:** Binisha Joshi, Yae Na Chun, Joyita Chakraborty, George Pandarakalam Thomas, Paul Bolaji, Mohammad Sarwar Khan Tharin, Ruth Adeyeye, Elizabeth Adeyeye, Sarah Azeta

**Affiliations:** 1 Internal Medicine, Dorset County Hospital, Dorchester, GBR; 2 Stroke Medicine, Dorset County Hospital, Dorchester, GBR; 3 General Medicine, Dorset County Hospital, Dorchester, GBR; 4 Internal Medicine, Colchester Hospital, East Suffolk and North Essex NHS Foundation Trust, Colchester, GBR; 5 Internal Medicine, University College London, London, GBR; 6 Internal Medicine, Imperial College London, London, GBR

**Keywords:** atrial fibrillation, cardiac monitoring, cardioembolic stroke, cost-effective monitoring, embolic stroke of undetermined source (esus), implantable loop recorder, personalized monitoring strategy (tias), stroke recurrence prevention

## Abstract

Atrial fibrillation (AF) is a significant contributor to cardioembolic stroke and often remains undiagnosed in cryptogenic or embolic strokes of undetermined source (ESUS). Extended cardiac monitoring has become a crucial strategy for enhancing post-stroke AF detection. Landmark trials such as EMBRACE, ASSERT, and CRYSTAL-AF have demonstrated higher AF detection rates with longer or implantable devices compared to standard short-term electrocardiograms (ECGs).

This narrative review examines selected randomised controlled trials (RCTs), extensive prospective studies, meta-analyses, and guideline statements, while excluding case reports, small single-centre studies, and reports without AF detection endpoints on extended cardiac monitoring. It provides a detailed comparison of different monitoring strategies alongside their relative cost-effectiveness. It primarily assesses cost per quality-adjusted life year (QALY), direct healthcare expenditure, and, where available, indirect costs such as readmissions, complications, accessibility, and benefits.

The review also introduces the innovative "TIAS" strategy for cardiac monitoring following stroke, which stands for Timing, Individualised, Amalgamated, and Stepwise approach. It aims to assist clinicians in developing an evidence-based, cost-effective plan for cardiac monitoring.

By employing customised monitoring strategies that account for each patient's risk factors, stroke subtype, and healthcare setting, the TIAS approach aims to improve AF diagnosis and reduce the likelihood of stroke recurrence, while acknowledging limitations such as heterogeneity across trials, uncertainty about AF burden thresholds, and challenges in real-world implementation across variable healthcare systems.

## Introduction and background

Stroke remains a significant global health burden and is among the leading causes of death and long-term disability worldwide. Recent estimates indicate over 12 million new cases and 6.5 million deaths each year, with a rising incidence in low- and middle-income countries [1,2]. Approximately 25% of ischaemic strokes are cardioembolic, mainly caused by atrial fibrillation (AF), which is projected to affect more than 60 million people worldwide by 2050 [3]. AF-related strokes are generally more severe and carry a higher risk of recurrence and disability.

Despite advances in imaging and diagnostics, up to 40% of ischaemic strokes remain cryptogenic. Cryptogenic strokes are strokes without an identifiable cause [4]. Many are classified as embolic strokes of undetermined source (ESUS). This concept was proposed to distinguish them from other cryptogenic strokes by requiring the absence of significant large artery stenosis, major cardioembolic sources, or lacunar pathology [5]. Detecting paroxysmal or subclinical AF is a key challenge, as it often remains undiagnosed at the initial event [6]. Conventional monitoring, such as a 24-72-hour Holter monitor, has limited sensitivity for these intermittent arrhythmias but is widely used because it is inexpensive, accessible, and feasible, even in resource-limited stroke units.

Over the past decade, landmark trials have demonstrated the significance of extended cardiac monitoring. The ASSERT trial revealed that subclinical atrial tachyarrhythmias detected by implants more than doubled the risk of stroke [3]. The EMBRACE trial showed that 30-day monitoring detected AF in 16.1% of patients compared to 3.2% with 24-hour Holter monitoring [7]. Similarly, the CRYSTAL-AF trial identified AF in 12.4% of patients within 12 months using implantable monitors versus 2.0% with traditional methods [8]. Emerging wearable and artificial intelligence (AI)-based solutions are encouraging, although their cost and practicality are still under investigation.

Starting oral anticoagulation in confirmed AF can decrease stroke risk by up to 64% [9], but due to bleeding risks, treatment should be limited to confirmed diagnoses. Transitioning from external to implantable devices is usually considered for recurrent cryptogenic stroke, ESUS with high AF suspicion, or inconclusive external monitoring.

Our proposed "TIAS" framework is a structured and pragmatic approach to guide cardiac monitoring after stroke. It is based on four key pillars: timing, individualised approach, amalgamated modalities, and a stepwise escalation strategy.

The TIAS algorithm is a conceptual framework based on landmark trials and guideline consensus, although it has not yet been prospectively validated. We selected these four elements because they directly address current gaps: optimal timing of monitoring, personalised escalation based on risk (CHA₂DS₂-VASc, AF-ESUS scores), integration of multiple modalities, and a pragmatic stepwise escalation that considers system-level constraints.

**Figure 1:**
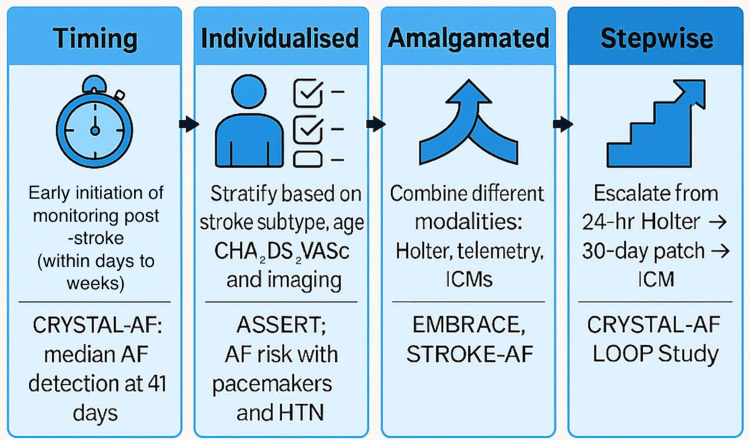
TIAS Framework for Post-stroke Atrial Fibrillation (AF) Detection The TIAS algorithm outlines a four-step approach to post-stroke AF detection: early Timing of monitoring, Individualised risk stratification, Amalgamated use of modalities, and Stepwise escalation from short to long-term monitoring, supported by studies such as CRYSTAL-AF, ASSERT, EMBRACE, and LOOP. Diagram created using data from [3,7,8].

These four pillars were prioritised over other potential elements because they directly address key barriers identified in the current evidence. This narrative review examines the evidence supporting prolonged monitoring, compares available strategies, and outlines how the TIAS framework can guide clinicians in implementing a cost-effective, clinically meaningful approach to AF detection following stroke, as illustrated in Figure 1.

## Review

Modalities of cardiac monitoring after stroke

Inpatient Cardiac Monitoring

Early inpatient cardiac monitoring is crucial for detecting AF in patients admitted with ischaemic stroke, especially when there is no previous history of AF. It is standard practice to monitor a patient’s heart rhythm using telemetry or a continuous electrocardiogram (ECG) during the first 24-72 hours of their acute hospital stay. This allows clinicians to identify paroxysmal or transient episodes of AF that might otherwise go unnoticed.

Timely detection of AF during hospitalisation is crucial, as it directly affects treatment choices, especially the initiation of oral anticoagulation to lower the risk of recurrent stroke. Research has shown that early rhythm monitoring in stroke units increases AF detection rates and reduces delays in initiating secondary prevention measures [6,10].

Inpatient monitoring provides a controlled environment for risk assessment, allowing for a thorough evaluation of high-risk individuals prior to discharge. While long-term methods provide continuous surveillance, prompt inpatient assessment remains crucial. Standard protocols can improve AF detection, reduce missed diagnoses, and enable timely stroke prevention. Guidelines such as those of the European Stroke Organisation (ESO) 2023 and the American Heart Association/American Stroke Association (AHA/ASA) 2021 recommend 24-72 hours of inpatient telemetry for acute ischaemic stroke [11,12], striking a balance between effectiveness and practicality. However, limitations include restricted sensitivity, false positives from artefacts, and resource demands, which strain busy or low-income stroke units. Patients with known AF on anticoagulation may not benefit from prolonged telemetry, highlighting the need for tailored approaches.

Ambulatory Monitoring

Ambulatory cardiac monitoring is crucial for detecting post-stroke AF, particularly after the inpatient phase. These non-invasive devices allow prolonged rhythm monitoring in outpatient settings, capturing intermittent arrhythmic episodes that might be missed during short-term hospital observation. There are different types of ambulatory monitoring, which include Holter monitoring (usually 24 hours to 7 days), patch monitors (such as Ziopatch, up to 14 days), extended external loop recorders (ELRs, up to 30 days), and mobile cardiac telemetry, which can also detect activity for up to 30 days.

The Holter electrocardiogram system provides continuous monitoring for 24-48 hours, with extended versions available for up to 7 days. It is user-friendly and widely accessible, making it a common first-line diagnostic tool. The detection rate of AF after a stroke is approximately 9.4% with 24-hour Holter monitoring, increasing to 12.7% with seven-day monitoring [1]. A meta-analysis reported a pooled detection rate of 10.7% (95% CI: 5.6-17.2%) compared to 5.1% (95% CI: 3.8-6.5%) during inpatient telemetry [1]. However, Holter monitors may miss paroxysmal AF, particularly when episodes are infrequent or asymptomatic [13].

Patch monitors, such as Ziopatch, are single-use wearable adhesive patches. They provide continuous single-lead ECG recordings for up to 14 days (sometimes extended in newer versions). Patients wear them like a sticker, and then the patch is mailed back to the company, where a full report is generated [14].

ELRs, also known as automatic event recorders, offer extended monitoring periods, typically ranging from 7 to 30 days. These devices can be either patient-activated or fully automatic. Their longer duration enhances diagnostic yield, particularly for asymptomatic or brief episodes of AF lasting 30 seconds or more. Detection rates increase with monitoring time: studies show rates of 14.3% at 4 days and up to 20% at 30 days [6,8].

Mobile cardiac telemetry is similar to ELR in terms of monitoring duration, lasting up to 30 days and being wearable. It provides real-time, continuous ECG monitoring with wireless data transmission, usually via cellular networks. It detects and automatically transmits arrhythmia events (AF, pauses, tachycardia, bradycardia) to a monitoring centre 24/7. It has higher sensitivity in detecting silent and asymptomatic AF compared with ELR, although it appears to be more expensive [14].

EMBRACE confirmed the benefits of extended monitoring, showing that a 30-day ELR detected AF in 16.1% of cryptogenic stroke patients versus 3.2% with a 24-hour Holter monitor [6]. Extended monitoring increased the detection of AF, leading to more effective anticoagulation. Specifically, AF lasting ≥30 seconds was found in 16.1% of the event monitor group compared to 3.2% in the control, highlighting the advantages of longer monitoring. However, the effectiveness of ELR can be limited by patient compliance, especially with manual activation models. Fully automated ELRs could overcome these issues, particularly for silent or infrequent AF.

Comparative data show that ELRs typically have higher sensitivity (~80%) and specificity (~90%) than Holters, which are limited by lower sensitivity (<50%) for paroxysmal AF [15]. Notably, the ≥30-second threshold used in many trials lacks uniform clinical significance: ASSERT linked episodes >6 minutes to stroke risk [16], while TREND suggested higher risks with burdens >5.5 hours per day [17]. This variability complicates anticoagulation decisions. Practical barriers also exist, particularly in LMICs, where limited device availability, follow-up capacity, and patient education may hinder effective use [18]. Few trials extend beyond 30 days of external monitoring, leaving a gap in evidence for detection strategies between one month and the multi-year surveillance provided by implantable cardiac monitors.

By adopting a tiered approach in clinical practice, healthcare professionals can implement a practical and evidence-based strategy. This involves starting with short-term Holter monitoring and then escalating to ELRs if initial tests yield inconclusive results. This method aligns with the stepwise principle of the TIAS framework, ultimately leading to improved secondary stroke prevention through earlier diagnosis and treatment of AF. The complete list of ambulatory monitoring devices is summarised in Table 1.

**Table 1:** Different Ambulatory Monitoring Devices Created using data from ref. [6,8,13-17].

How it records	Typical wear	Data transfer	Triggering	AF detection (post-stroke/cryptogenic)	Strengths	Limitations
Holter (24–48 h; up to 7 d)	Continuous ECG (1–3 leads)	1–2 days (some up to 7 days)	Stored, reviewed after return	No activation needed	~3–5% at 24–48 hours; ~8–12% by 7 days	Widely available; low cost; simple
External loop recorder (ELR)	Continuous looping buffer; saves when triggered (auto + patient)	7–30 days	Usually, periodic uploads or post-wear downloads	Auto-trigger ± patient button	~14–20% at 30 days (EMBRACE ~16%)	Longer duration; captures asymptomatic AF if auto-trigger is enabled
Mobile cardiac telemetry (MCT)	Continuous ECG with real-time algorithmic detection	~14–30 days	Live cellular transmission to a 24/7 monitoring center	Automatic; optional patient mark	Often higher than ELR over the same duration (≈20–25% in similar cohorts)	Captures asymptomatic AF; near real-time alerts; clinician action can be faster
Patch monitor (e.g., Zio Patch)	Continuous single-lead ECG (adhesive patch)	10–14 days (sometimes longer)	Stored; analyzed after return	No activation needed	~5–15% at 14 days (cohort-dependent)	Comfortable; water-resistant; high wear-time compliance; no wires

Implantable Devices and Cardiac Devices Using SMART Technology

Insertable cardiac monitors (ICMs), or implantable loop recorders (ILRs), are small devices placed under the skin in the chest that provide continuous ECG monitoring for three to five years [6,8]. They detect arrhythmias using preset algorithms, especially useful for patients with infrequent, unexplained, or asymptomatic cardiac events. Models include Medtronic Reveal LINQ, Abbott Confirm Rx, BIOTRONIK BioMonitor III, and Boston Scientific LUX-Dx, each with features such as smartphone connectivity, remote monitoring, advanced detection, and extended battery life [6,8]. False-positive rates vary, with newer algorithms improving specificity, though some studies report rates of 40-55%, mainly from misinterpreting premature contractions [19,20].

Patient selection for ICM implantation typically relies on risk stratification. Candidates include those with cryptogenic stroke (especially under 60 years), recurrent unexplained syncope, or a high clinical suspicion of paroxysmal AF despite negative short-term monitoring. Conversely, smart devices may be more suitable for lower-risk patients or individuals undergoing opportunistic screening rather than formal diagnostic monitoring [19,21]. In cryptogenic stroke and ESUS, ICMs are particularly valuable for detecting subclinical AF that shorter monitoring often fails to identify. Multiple randomised trials confirm this advantage [10]. The CRYSTAL-AF trial, for example, demonstrated AF detection in 12.4% of patients at 12 months with ILR monitoring compared with only 2.0% in those receiving standard care [22]. Observational data suggest this yield increases to nearly 30% by three years [6,8].

ICMs have favourable safety profiles with low complication rates. Minor events include local infection, device migration, or small haematomas, while serious complications requiring removal remain under 1%. Their diagnostic yield has immediate therapeutic benefits. A systematic review and meta-analysis showed that ICMs detect more AF, increase anticoagulation, and may lower stroke rates [8]. Guidelines suggest considering anticoagulation when device-detected AF exceeds 24 hours or AF burden is ≥0.5-1%, though thresholds are evolving. The LOOP study questioned the clinical impact of widespread ILR use, finding no significant stroke reduction compared with usual care [23]. Limitations involved a mostly asymptomatic population, short follow-up, and issues of generalisability to higher-risk stroke survivors. These findings suggest very short, device-detected AF episodes may have less prognostic significance than longer, sustained arrhythmia [23].

Advances in consumer-grade cardiac technologies have opened new opportunities for AF detection, especially in patients with cryptogenic stroke who remain undiagnosed after standard inpatient or outpatient monitoring. Devices such as handheld ECG recorders, patch monitors, and wearable smartwatches now enable scalable, patient-centred rhythm monitoring in outpatient or community settings.

Smartphone-compatible ECG platforms, such as KardiaMobile, have been validated against clinical-grade ECGs and offer real-time heart rhythm recordings through patient activation [20]. Similarly, the Apple Watch, utilising photoplethysmography and single-lead ECG technology, has shown effectiveness in detecting arrhythmias. In the Apple Heart Study, involving over 400,000 participants, smartwatch-detected irregular rhythms were confirmed as AF in 34% of users with notifications, supporting the device's potential for large-scale screening [24].

Although most of these devices offer intermittent, patient-activated monitoring, they are beneficial for lower-risk patients or as a supplement to formal monitoring methods. Their convenience and accessibility appeal to younger individuals or those in remote areas where traditional ECG monitoring is difficult.

Both the ESO and UK NICE (National Institute for Health and Care Excellence) guidelines endorse using smart wearable technologies for certain stroke patients, especially when traditional methods produce inconclusive results or are unavailable [21]. Limitations include dependence on user engagement, brief monitoring durations, potential artefacts, and the lack of continuous automated detection.

Adherence is key. While ICMs provide automated monitoring, smart devices rely heavily on patient compliance. Research shows adherence to wearables drops to 60-80% after a year, lowering diagnostic efficacy [24,25]. Nonetheless, smart devices are appealing in low-risk or resource-limited settings due to their affordability and non-invasiveness. When these smart devices are included in a personalised, step-by-step approach, they offer a valuable supplement to formal monitoring. Their function aligns well with the TIAS framework, especially under the "Individualised" and "Stepwise" pillars, which support AF detection in situations where traditional methods might fall short.

Emerging technologies are refining platforms. AI-based arrhythmia detection in wearables and ICM algorithms improves specificity and reduces false positives, but prospective validation is limited [26,27]. Future research should examine long-term AF outcomes from smart devices, determine optimal anticoagulation thresholds, and explore combined wearable and ICM strategies to maximise detection and control costs.

In summary, ICMs offer unmatched long-term monitoring for high-risk patients such as stroke survivors, but their cost, invasiveness, and false-positive burden remain barriers to universal use. Smart devices, while less accurate, are increasingly relevant for opportunistic screening and may complement ICMs in tiered monitoring strategies. Achieving the right balance between accuracy, cost, adherence, and equity will require further comparative trials and health-economic analyses. Table 2 summarises various cardiac monitoring methods, including inpatient monitoring, ambulatory monitoring, implantable devices, and smart technology-based monitoring.

**Table 2:** Different Modalities of Cardiac Monitoring After Stroke Comparison of different cardiac monitoring modalities used after stroke, detailing their monitoring duration, AF detection rates, supporting evidence, advantages, limitations, and optimal clinical use cases. Modalities include inpatient telemetry, Holter monitoring, ELR, and ICM. AF: atrial fibrillation, ELR: external loop recorder, MCT: mobile cardiac telemetry, ICM: insertable cardiac monitor. Created using data from [1,6,8,19,22,23].

Modality	Monitoring duration	AF detection rate	Key trial evidence	Advantages	Limitations
Inpatient telemetry	24–72 hours	~5.1%	Meta-analysis	Immediate availability; useful in the acute phase	Short duration; may miss paroxysmal AF
Holter monitor (24–48 hours)	1–2 days (up to 7 days)	9.4% (24 hours), up to 12.7% (7 days)	Meta-analysis	Widely available; non-invasive	Low sensitivity for intermittent AF
ELR/MCT/Ziopatch	7–30 days	Up to 20%; 16.1% (EMBRACE)	EMBRACE	Longer duration; higher yield for paroxysmal AF	Requires patient compliance (if not auto); external device may be inconvenient
Implantable cardiac monitor (ICM)	Up to 3–5 years	12.4% at 12 months; ~30% at 3 years	CRYSTAL-AF, LOOP	Longest monitoring; automatic; ideal for silent/infrequent AF	Invasive; costly; false-positive risk (~55%)

Comparative efficacy of monitoring modalities

Detection Rates by Duration and Device Type

The ability to detect AF after a stroke depends largely on both monitoring duration and device type. Short-term methods such as 24-48-hour Holter monitors detect AF in only 2-5% of cases [1]. Extending monitoring significantly improves detection: 7-30 days increases the detection rate to 5-15% [13]. Mobile cardiac telemetry (MCT) worn for 14-30 days achieves detection rates of 10-15%, especially in symptomatic patients [14]. A 2023 study found that 14-day patch ECG monitors (e.g., ZioPatch) outperformed Holters by enhancing diagnostic accuracy and reducing the need for repeat testing [28].

The EMBRACE trial demonstrated that longer monitoring is more effective: AF was detected in 16.1% of patients using a 30-day external loop recorder compared to 3.2% with a 24-hour Holter (absolute difference 12.9%, 95% CI 8.0-17.6; P < 0.001) [6]. A 2023 meta-analysis found no significant difference in detection rates between MCT (15.3%; 95% CI 5.3-29.3%), ELRs (16.2%; 95% CI 0.3-24.6%), and implantable monitors (P = 0.97) in cryptogenic stroke patients [1], indicating similar effectiveness for extended non-invasive monitoring. However, patch monitors showed better compliance and accuracy compared with Holters, while MCT may more reliably detect symptomatic events than ELRs [14].

ICMs extend monitoring up to three years and consistently surpass external devices in detecting silent or intermittent AF. The CRYSTAL-AF trial showed AF detection in 12.4% of patients at 12 months with ICMs compared to 2.0% with routine care (HR 7.3; 95% CI 2.6-20.8; P < 0.001) [10], while STROKE-AF reported 21.7% detection at three years versus 2.4% with standard care (HR 10.0; 95% CI 5.2-19.3; P < 0.001) [29]. Rates are higher in ESUS than in other cryptogenic strokes, reflecting underlying atrial pathology [5]. Although invasive and costly, ICMs offer unmatched sensitivity in high-risk groups.

The clinical significance of AF depends on detection, burden, and duration. The LOOP study reported that AF episodes lasting more than six minutes were linked to a higher stroke risk [23]. Early-detected AF often has a greater burden, while late-detected AF may indicate a lower thromboembolic risk. Growing evidence suggests AF may be a marker of atrial cardiopathy rather than a direct cause. In ASSERT, only 8% of stroke patients had AF detected within 30 days, whereas in CRYSTAL-AF, many episodes appeared weeks to months after the stroke [23,10]. This suggests atrial vulnerability itself may predispose to embolism, with AF acting as a surrogate marker. Therefore, monitoring strategies could benefit from including biomarkers of atrial cardiopathy alongside rhythm surveillance [5].

Importance of Timing: Earlier Monitoring Yields Higher Detection

Timing and duration are both crucial. Monitoring within days of a stroke significantly improves AF detection compared with a delayed start. A meta-analysis found AF in 15% of patients when monitoring began within 7 days versus 5% when started after 30 days [30]. EMBRACE also showed that early 30-day monitoring detected AF in 16.1% compared with 3.2% using a standard 24-hour Holter [6]. Early detection allows for anticoagulation, which may reduce recurrence, but no trial has definitively shown that earlier monitoring directly decreases recurrent stroke, leaving a vital evidence gap [31].

Practical challenges restrict early initiation, especially in resource-limited settings. Barriers include device availability, staffing, and patient instability during the acute phase. Signal artefacts and motion interference further hinder interpretation in acute care, particularly with wearables such as smartwatches [20,32-34].

Modelling of economic data suggests that early monitoring could be cost-effective. Immediate ICM implantation has been shown to reduce Medicare payments by $3683-4070 per patient and out-of-pocket costs by $1425-1503 when compared with delayed monitoring [35]. Additionally, early patch monitoring has demonstrated favourable incremental cost-effectiveness, with an estimated value of approximately $36,000 per quality-adjusted life year (QALY) gained [36].

Emerging trials have also supported the aims of earlier and extended monitoring, particularly the earlier start of anticoagulation. There is evidence of safety, non-inferiority, and proven superiority of an early start to anticoagulation if AF is detected soon after stroke. The recent trials OPTIMAS, TIMING, and ELAN, and the CATALYST meta-analysis, validate this (early start of anticoagulation as early as 48 hours to 4 days) [37-39]. This further lends credence to the overall aim of early monitoring after stroke.

SAFFO (NCT04660747) and FIND-AF 2 (NCT04371055) are investigating advanced external monitoring in ESUS, while ARCADIA (NCT03192215) assesses anticoagulation in atrial cardiopathy without AF [40-42]. Their findings will clarify whether improved detection leads to meaningful reductions in recurrent stroke.

AF burden and clinical significance

Are all detectable AF episodes significant? The duration is crucial. Research indicates that very brief episodes (<30 seconds) may not pose the same stroke risk as longer, sustained AF [6]. In ASSERT, only subclinical AF lasting over 6 minutes was linked to a 2.5-fold increase in stroke risk [6], and the LOOP study also associated episodes over 6 minutes with a higher risk [23]. AF burden, defined as the total time spent in AF, is increasingly recognised as clinically relevant. The TREND study found that burdens exceeding 5.5 hours per day increased stroke risk [31], while GUARD-AF showed that a greater AF burden correlated with a higher risk, with those exceeding 5% burden being particularly vulnerable [35].

Despite these insights, the threshold for anticoagulation remains under debate. Guidelines still define AF as ≥30 seconds but reconcile this with newer data by recommending anticoagulation for device-detected AF of longer duration (e.g., >5.5 hours) or in patients with higher CHA₂DS₂-VASc scores [5,19]. For device-detected AF, lower thresholds such as >6 minutes often lead to consideration, whereas in clinically apparent AF, any paroxysmal episode may require treatment if the overall risk is high [19]. Patient-specific factors, especially CHA₂DS₂-VASc ≥2 in men or ≥3 in women, can justify anticoagulation even for brief episodes [5]. Variability in study cut-offs, ranging from 6 minutes (ASSERT, LOOP) to 5.5 hours (TREND) or even 24 hours, creates uncertainty and inconsistent practice, requiring clinicians to weigh both burden and risk scores until stronger evidence is available.

Ongoing trials such as DEFINE AFib, ARTESIA, and NOAH-AFNET 6 aim to refine burden-based thresholds, although results so far indicate that anticoagulation reduces stroke risk but increases bleeding [31]. The clustering of AF episodes may also be significant; multiple closely spaced events seem more prognostic than isolated ones, with some studies showing a threefold increase in stroke risk shortly after clustered episodes [6].

**Figure 2:**
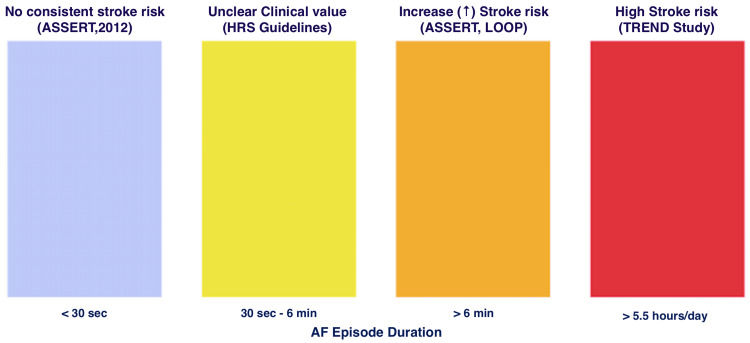
Clinical Significance of Atrial Fibrillation Episode Duration With Key Evidence Graphical representation of the clinical significance of AF episode duration based on key trials. Stroke risk is minimal for episodes <30 seconds (ASSERT), uncertain between 30 seconds and 6 minutes (HRS Guidelines), and increases progressively with duration >6 minutes (ASSERT, LOOP) and >5.5 hours/day (TREND study). Created using data from [6,11,23,35].

Current evidence has limitations, including reliance on observational data and device algorithms prone to false positives [6,23]. Nonetheless, the clinical message is clear: monitoring should focus less on individual short events and more on AF burden and context, enabling better risk stratification and personalised anticoagulation, particularly in asymptomatic patients. The graphical representation of the clinical significance of AF based on key trials is illustrated in Figure 2.

Cost-effectiveness and global availability

Cost Comparison: Holter vs. ELRs vs. ICMs

The cost of cardiac monitoring devices varies greatly depending on duration, invasiveness, and diagnostic effectiveness. Holter monitors, which provide non-invasive ECG recordings over 24 hours to 7 days, are the most affordable option. In the United States (US), the cost is roughly $600, while in the United Kingdom (UK), it is about £210 [43].

ELRs, which enable longer real-time monitoring (up to 30 days) and event-triggered detection, are somewhat more expensive, averaging $743 in the US (including professional and facility fees) and £250-£800 in the UK [44].

ICMs, such as the Medtronic Reveal LINQ, offer continuous monitoring for up to three years and deliver the highest diagnostic yield, especially for intermittent or asymptomatic AF. However, this comes with a significant cost: approximately $6469 in the US (device and implantation) and £2300 in the UK [35]. These figures are based on reimbursement data reported in studies from 2012, 2016, and 2022, the most recent available. No adjustments for inflation were made, as costs vary by insurance provider and are not consistently reported in the literature.

Insurance and Public Healthcare: US vs. UK

In the US, most insurance plans provide partial coverage, but patients often pay 20% or more out of pocket depending on policy, facility, and diagnosis [36]. Costs are higher for out-of-network care or hospital-based services. Cost-effectiveness in the US is generally compared against a threshold of $150,000 per QALY gained [45].

In the UK, cardiac monitoring is provided through the NHS, with national tariffs covering devices such as the Reveal LINQ. Out-of-pocket costs are minimal, and interventions are generally considered cost-effective if they fall between £20,000 and £30,000 per QALY, according to NICE guidance. By contrast, many low- and middle-income countries (LMICs) lack formal QALY thresholds and often rely on the World Health Organisation (WHO) "rule of thumb," which considers interventions costing less than one to three times GDP per capita per QALY gained to be cost-effective.

Access and Equity in Low- and Middle-Income Countries (LMICs)

Access to advanced monitoring in LMICs remains limited. Even basic ECG services may be constrained by shortages of equipment, consumables, electricity, and trained personnel [32]. Urban centres might offer advanced diagnostics, but these are often prohibitively expensive and must be paid for out of pocket.

The adoption of wearable systems, including patch devices and smartwatches, has shown promising potential. A recent systematic review reported pooled sensitivity and specificity above 96% for both ECG patches and photoplethysmography (PPG)-based smartwatches [46]. Studies in LMICs demonstrate feasibility, but questions remain about long-term cost-effectiveness and sustainability [47]. Innovative financing strategies, including public-private partnerships and subsidised device programmes, have been proposed as methods to expand access in resource-limited settings.

Implantable monitors are rarely utilised in LMICs due to cost, infrastructure gaps, and workforce shortages for implantation and follow-up [33]. Insurance coverage is inconsistent, with patients often facing shared out-of-pocket expenses. Reimbursement and funding constraints emphasise the need for stronger, sustainable cardiovascular health systems supported by international and local investment [32].

Balancing Yield and Cost: Is More Always Better?

Holter monitors are inexpensive and widely accessible but often miss paroxysmal or asymptomatic AF due to limited monitoring periods. Extended monitoring enhances detection, especially in cryptogenic and ESUS subgroups, where the yield is highest. In EMBRACE, 16.1% of cryptogenic stroke patients were diagnosed with AF using 30-day recorders compared with 3.2% with 24-hour Holters [6]. Although longer monitoring involves higher initial costs (≈£1000), the cost per QALY of £20,000 remains within the NICE threshold for cost-effectiveness.

ICMs such as the Reveal LINQ detect AF three to six times more often than short-term or 30-day methods, as demonstrated in CRYSTAL-AF, PER DIEM, and LOOP. The LOOP study also indicated a threefold increase in anticoagulation initiation with ICM monitoring [23]. The burden of AF thresholds partly accounts for this, as higher-burden episodes are more likely to trigger anticoagulation, thereby enhancing cost-effectiveness compared with short, low-burden episodes.

ICMs are expensive (≈£6000, with cost per QALY estimates ranging £40,000-50,000), but they could be cost-effective for high-risk groups such as younger patients, those with ESUS, elevated CHA₂DS₂-VASc scores, and low bleeding risk. Additionally, indirect costs such as preventing recurrent hospitalisations, long-term disability care, and stroke-related productivity loss are often overlooked in models, but including them would likely make prolonged monitoring more favourable [48]. From a Medicare and patient perspective, several models indicate that early ICM placement after stroke results in long-term savings compared with repeated short-term monitoring [35].

MCT offers a middle-ground option, enabling longer monitoring periods with real-time feedback. It is less invasive than ICMs but more expensive than ELRs. This presents a key dilemma: balancing diagnostic advantages with resource expenditure. Additionally, estimates of cost-effectiveness remain uncertain, as they largely depend on assumptions regarding anticoagulation adherence, stroke recurrence risk, and patient behaviour in real-world settings.

Cardiac monitoring after stroke requires a balance between diagnostic yield, cost, and patient context. While extended monitoring is more expensive, it detects significantly more AF, especially in ESUS patients. Holters are suitable for low-risk or resource-limited settings, while ILRs and MCT may be cost-effective in high-risk groups. More research is needed to provide real-world cost-effectiveness data across diverse health systems, particularly in LMICs, where evidence is sparse. In the right patient, even costly interventions may be justified if they prevent recurrent, disabling strokes.

The TIAS algorithm for the stepwise use of cardiac monitoring for AF in patients with stroke

Introducing *the TIAS Strategy*

Based on this extensive review of landmark trials, we therefore propose a novel "TIAS" framework, which is a structured and pragmatic approach to guide cardiac monitoring after stroke. It is based on four key pillars: timing, individualised approach, amalgamated modalities, and a stepwise escalation strategy.

**Figure 3:**
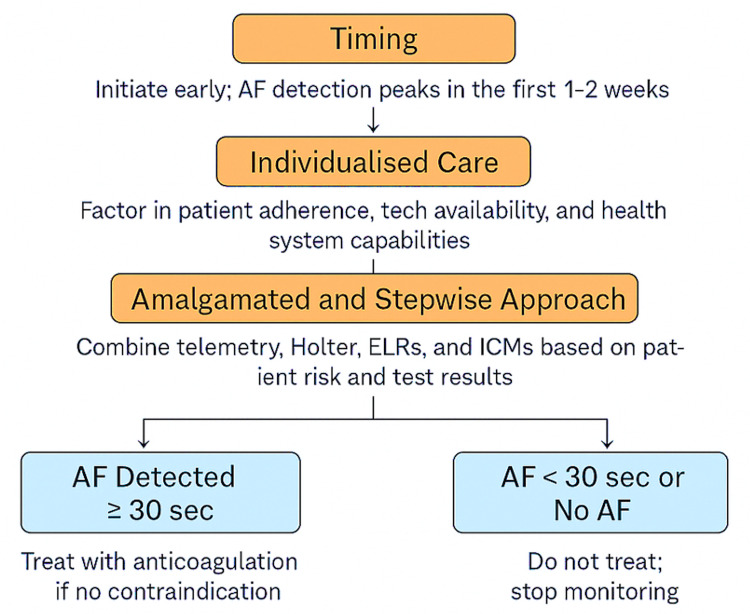
TIAS Strategy for Stepwise Use of Cardiac Monitoring for Atrial Fibrillation in Patients With Stroke Flowchart showing TIAS strategy: early, tailored, stepwise monitoring for AF post-stroke. Anticoagulated if AF is ≥30 seconds; stop if it is <30 seconds or no AF. Developed a flowchart utilising data from [30].

The TIAS algorithm is a conceptual framework developed from landmark trials and guideline consensus, although it has not yet been prospectively validated. The visual presentation of the TIAS strategy for step-by-step use of cardiac monitoring for AF in patients with stroke is shown in Figure 3.

The TIAS Strategy

A comprehensive strategy for AF detection after stroke must combine timing, personalised care, and a step-by-step, integrated approach.

Timing: Early monitoring within the first 72 hours is essential, as AF detection is most likely to occur within the first one to two weeks, especially in cryptogenic and ESUS cases [30].

Individualised care: Monitoring should be tailored to the individual, considering stroke subtype, age, comorbidities, CHA₂DS₂-VASc score, and AF-ESUS score, as well as adherence potential, comfort with technology, and resource availability. Patients with a CHA₂DS₂-VASc score of 2 or higher in men (or 3 or higher in women) or an AF-ESUS score of 3 or more are considered high risk and should be prioritised for extended or implantable monitoring. Practical tailoring also considers digital literacy, device tolerance (such as skin irritation from patches), and patient preferences.

Amalgamated and stepwise approach: The stepwise approach begins with inpatient telemetry (24-72 hours), then progresses to Holter monitoring (48 hours to 7 days), and finally utilises external loop recorders (ELRs, 14-30 days). If results are inconclusive, long-term insertable cardiac monitors (ICMs, one to three years) are recommended, particularly for high-risk groups. This progression is supported by the EMBRACE, CRYSTAL-AF, and PER DIEM trials [23,30,31,36]. Detection rates increase at each step: 24-72-hour telemetry (2-5%), 7-day Holter (10-12%), 30-day ELR (16-20%), and ILRs over three years (30-35%). Stopping rules suggest no further escalation if AF is not detected after ≥30 days of external monitoring or ≥12 months of ILR in patients without recurrent embolic events. Importantly, real-world adherence and diagnostic yield may be lower than in trial settings, emphasising the need for continual evaluation of this framework in clinical practice. The 30-second threshold for AF was established based on expert consensus and trial standards; however, increasing evidence suggests that longer or clustered episodes may better predict thromboembolic risk. Consequently, the TIAS framework now includes AF burden and recurrence patterns in monitoring and decision-making regarding anticoagulation.

Monitoring Strategy for ESUS (Embolic Stroke of Undetermined Source) in the TIAS Strategy

All patients experiencing an ischaemic stroke who are not known to have AF should undergo short-term cardiac monitoring early during their hospital stay to detect AF and guide anticoagulation treatment. The first step is inpatient telemetry for 24 to 72 hours, which is the standard initial monitoring method during hospitalisation. This approach detects AF in about 2-5% of cases and is endorsed by the 2024 American College of Cardiology (ACC) Expert Consensus Decision Pathway [19]. It is straightforward, cost-effective, and accessible, including in LMICs.

If AF is not detected, short-term Holter monitoring (24-72 hours) should be carried out, ideally within one to two weeks. If no findings are observed, extended monitoring with a 7-30-day external loop recorder (preferably 30 days, as in the EMBRACE trial) is recommended. For high-risk patients without AF, implantable cardiac monitors (ICMs) are the next step, while smart wearable ECG devices may serve as alternatives in some cases or in low-resource settings. This protocol enhances the detection of AF, informs anticoagulation decisions, and reduces the risk of stroke recurrence.

**Figure 4:**
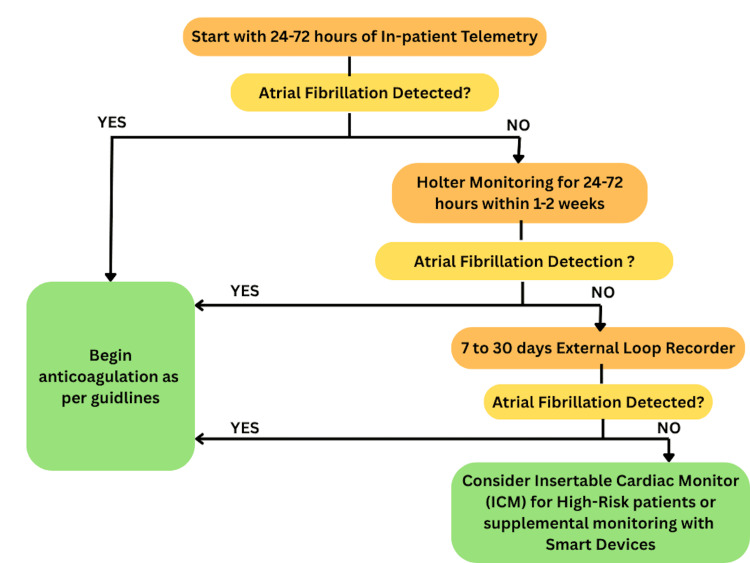
Algorithm for ESUS Patients Flowchart representing stepwise AF detection in ESUS: start with telemetry, then Holter, followed by ELR if needed. If no AF is detected, consider ICM (Intelligent Cardiac Monitoring) or intelligent monitoring. Start anticoagulation if AF is found. AF: atrial fibrillation, ESUS: embolic stroke of undetermined source, ELR: external loop recorder, ICM: insertable cardiac monitor. Created this flowchart using data from [19,30,32-34].

In LMICs, implementing these methods in daily settings encounters significant barriers related to accessibility and affordability, which are further exacerbated by infrastructure constraints that impede the deployment of ICMs and the ongoing use of ELRs [32,33]. Wearable devices offer promising, scalable options, but they require some familiarity with technology [34,32]. As healthcare technology and AI continue to advance, future strategies are likely to become more accessible and adaptable. The flowchart demonstrating this monitoring algorithm is presented in Figure 4.

Risk Stratification, Efficacy, and Cost Implications to Justify Extended Monitoring in the TIAS Strategy

Following initial telemetry, risk stratification should be performed using key clinical criteria such as age over 65, a history of prior transient ischaemic attack (TIA), evidence of left atrial enlargement on echocardiography, and a high CHA₂DS₂-VASc score.

Clinical models such as the AF-ESUS score can be used to determine whether more extensive monitoring is necessary. If no AF is detected on telemetry but the patient is considered high risk, then escalation to extended cardiac monitoring is suitable.

Research indicates that AF accounts for 10-30% of ESUS cases, with detection rates varying depending on the duration and method used [1]. A risk-based, stepwise approach enhances detection while conserving resources.

Practically, inpatient telemetry and Holter monitoring are affordable and generally accessible. For outpatient care, wearable or patch-based remote devices are suitable options; however, ICMs should be primarily used for high-risk patients or those with a history of recurrent cryptogenic strokes.

ELRs are used for 7 to 30 days, increasing the chances of detecting atrial fibrillation. The EMBRACE trial reported a 16.1% detection rate during 30-day monitoring [6], with detection often occurring within the first one to two weeks after a stroke [30,31]. These devices strike a balance between affordability and usefulness for medium-term rhythm assessment, although adherence may vary with manually triggered types. They improve outcomes, as demonstrated in EMBRACE, by increasing anticoagulation initiation and potentially reducing recurrent stroke risk by up to 70% with proper antithrombotic therapy [6,8,29].

For undetected AF, ICMs such as Medtronic Reveal LINQ provide long-term surveillance, detecting AF in up to 30% over three years, as demonstrated in the CRYSTAL-AF trial [22], with an average detection time of around 8-10 months compared with shorter times with extended external loop recorders [6,22,31]. Although ICMs are costly and require invasive implantation, they are suitable for individuals at high risk due to their improved long-term effectiveness in identifying AF and prompting anticoagulation, as shown by the LOOP and CRYSTAL-AF studies. Such interventions may reduce the risk of repeat strokes by up to 70% when combined with anticoagulant treatment [8,22,23,29]. Moreover, patient adherence tends to be higher due to the devices' fully automated functionality.

ICMs are suitable for high-risk patients over 65 with left atrial enlargement, prior stroke or TIA, and no contraindications to anticoagulation. Tools such as CHA₂DS₂-VASc, AF-ESUS score, and HAVOC score assist in evaluating and refining patient selection by considering factors such as age, previous TIA, left atrial dilation, vascular risk, and comorbidities [5,10,34]. These align with the 2023 ESO guidelines, which recommend extended rhythm monitoring for cryptogenic strokes [21]. The LOOP trial demonstrates that ICMs triple AF detection rates and increase anticoagulation initiation compared with standard care [23].

However, both ICMs and wearable devices carry the risk of overtreatment, as subclinical AF, such as short episodes under 30 seconds, often does not require anticoagulation. Insights from the LOOP and ASSERT trials indicate that only episodes lasting over 6 minutes significantly increase stroke risk, advising caution in treatment initiation based on detection [3,23,31].

Limitations and future recommendations of this study

This review adopts a narrative approach, drawing on key trials, guideline statements, and selected prospective studies to highlight post-stroke AF detection strategies. Unlike a systematic review, it does not comprehensively capture all available evidence, and some selection bias may be present. The aim is to provide a practical synthesis to inform clinical decision-making, while recognising that future systematic reviews and prospective studies will be necessary to validate and expand upon these findings. The TIAS framework is a conceptual approach and will need validation in real-life scenarios using prospective methods.

## Conclusions

AF is a significant yet frequently under-recognised cause of ischaemic stroke, particularly in cryptogenic and ESUS cases. Early detection of AF allows for timely anticoagulation treatment, thereby reducing the risk of recurrence. While extended monitoring methods such as ICMs have higher detection rates than short-term Holter monitors, not all AF episodes carry the same prognostic weight. Brief or low-burden AF may be less clinically significant, so treatment decisions should consider episode duration, overall burden, and individual risk factors rather than detection alone. In resource-limited settings, wearable devices and smartphone-based ECGs are promising and affordable options, although challenges related to accuracy, adherence, and confirmation persist. A stepwise approach starting with inpatient telemetry, advancing to external monitors, and reserving ICMs for higher-risk patients provides a practical and cost-efficient diagnostic pathway.

The TIAS algorithm presented here provides a conceptual framework to guide clinicians in decision-making. By integrating timing, personalised risk scores, combined modalities, and a gradual escalation process, it aims to optimise detection, stroke prevention, and resource utilisation. Although it has not yet been validated through prospective trials, it emphasises the importance of structured, patient-centred approaches that account for both clinical and system-level constraints. The main challenges ahead are determining thresholds for anticoagulation in subclinical AF and avoiding overtreatment of incidental findings. Advances in artificial intelligence and digital wearables might improve detection and risk assessment. However, improving outcomes will ultimately rely on prospective validation of frameworks such as TIAS to ensure that AF monitoring strategies are both clinically sound and practical in various healthcare environments.
